# Blood biomarkers of progressive atherosclerosis and restenosis after stenting of symptomatic intracranial artery stenosis

**DOI:** 10.1038/s41598-021-95135-y

**Published:** 2021-08-02

**Authors:** Melanie Haidegger, Markus Kneihsl, Kurt Niederkorn, Hannes Deutschmann, Harald Mangge, Christian Vetta, Michael Augustin, Gerit Wünsch, Simon Fandler-Höfler, Susanna Horner, Christian Enzinger, Thomas Gattringer

**Affiliations:** 1 Department of Neurology, Medical University of Graz, Auenbruggerplatz 22, 8036 Graz, Austria; 2 Division of Neuroradiology, Vascular and Interventional Radiology, Department of Radiology, Medical University of Graz, Graz, Austria; 3 Clinical Institute of Medical and Chemical Laboratory Diagnostics, Medical University of Graz, Graz, Austria; 4 Institute for Medical Informatics, Statistics and Documentation, Medical University of Graz, Graz, Austria

**Keywords:** Cerebrovascular disorders, Biomarkers

## Abstract

In-stent restenosis (ISR) represents a major complication after stenting of intracranial artery stenosis (ICAS). Biomarkers derived from routine blood sampling including C-reactive protein (CRP), neutrophil-to-lymphocyte ratio (NLR), platelet-to-lymphocyte ratio (PLR) and mean platelet volume (MPV) have been associated with progressive atherosclerosis. We investigated the role of CRP, NLR, PLR and MPV on the development of intracranial ISR and recurrent stroke risk. We retrospectively included all patients who had undergone stenting of symptomatic ICAS at our university hospital between 2005 and 2016. ISR (≥ 50% stenosis) was diagnosed by regular Duplex sonography follow-up studies and confirmed by digital subtraction angiography or computed tomography angiography (mean follow-up duration: 5 years). Laboratory parameters were documented before stenting, at the time of restenosis and at last clinical follow-up. Of 115 patients (mean age: 73 ± 13 years; female: 34%), 38 (33%) developed ISR. The assessed laboratory parameters did not differ between patients with ISR and those without (p > 0.1). While ISR was associated with the occurrence of recurrent ischemic stroke (p = 0.003), CRP, NLR, PLR and MPV were not predictive of such events (p > 0.1). Investigated blood biomarkers of progressive atherosclerosis were not predictive for the occurrence of ISR or recurrent ischemic stroke after ICAS stenting during a 5-year follow-up.

## Introduction

Atherosclerotic intracranial artery stenosis (ICAS) is an important cause of ischemic stroke and responsible for approximately 5–10% of all strokes in the western population^1^. Compared to other stroke subtypes, ICAS-related stroke is associated with a higher risk for recurrent transient ischemic attacks (TIA) or stroke^2–4^.

For secondary stroke prevention, the best treatment option (intracranial artery stenting vs. medical treatment only) for ICAS patients remained unclear over years^5^. Since the results of the SAMMPRIS study were published in 2011, aggressive medical therapy with dual antiplatelet agents and high-dose statin therapy has internationally been accepted as the gold standard for treating ischemic stroke caused by intracranial atherosclerotic disease^6^. In 2015, results from the VISSIT trial, which showed high rates of stroke and TIA in the best medical treatment plus stenting group, also supported aggressive medical management alone as optimal treatment for symptomatic ICAS^7^.

However, some patients continue to suffer from recurrent ischemic events despite best medical treatment. In such cases endovascular stenting of ICAS remains a treatment option. The high rates of in-stent restenosis (ISR, ≈ 30%) and recurrent ischemic events after intracranial artery stenting underscore the value of an individualized patient selection and follow-up regimen^1,2,8–10^.

In this context, biomarkers that are predictive for ISR after stenting of ICAS would help to identify patients unlikely to benefit from such therapy or needing intensified follow-up observation. Several studies investigated the impact of routine blood biomarkers as predictors for progressive atherosclerosis in different vascular beds (i.e. coronary arteries, carotids and iliac/femoral arteries)^10–14^. However, data on laboratory markers in ICAS patients are still lacking.

In these studies, mean platelet volume (MPV), neutrophil-to-lymphocyte ratio (NLR), C-reactive protein (CRP) and platelet-to-lymphocyte ratio (PLR) were associated with progressive atherosclerotic carotid artery stenosis (MPV, NLR), critical limb ischemia (CRP) and coronary artery disease (NLR, PLR)^11–16^.

We therefore aimed to investigate the value of such routine blood parameters for the prediction of ISR and recurrent ischemic events after stenting of symptomatic high-grade intracranial artery stenosis in a monocentric study with comparatively long follow-up.

## Methods

### Study design and participants

**Figure 1:**
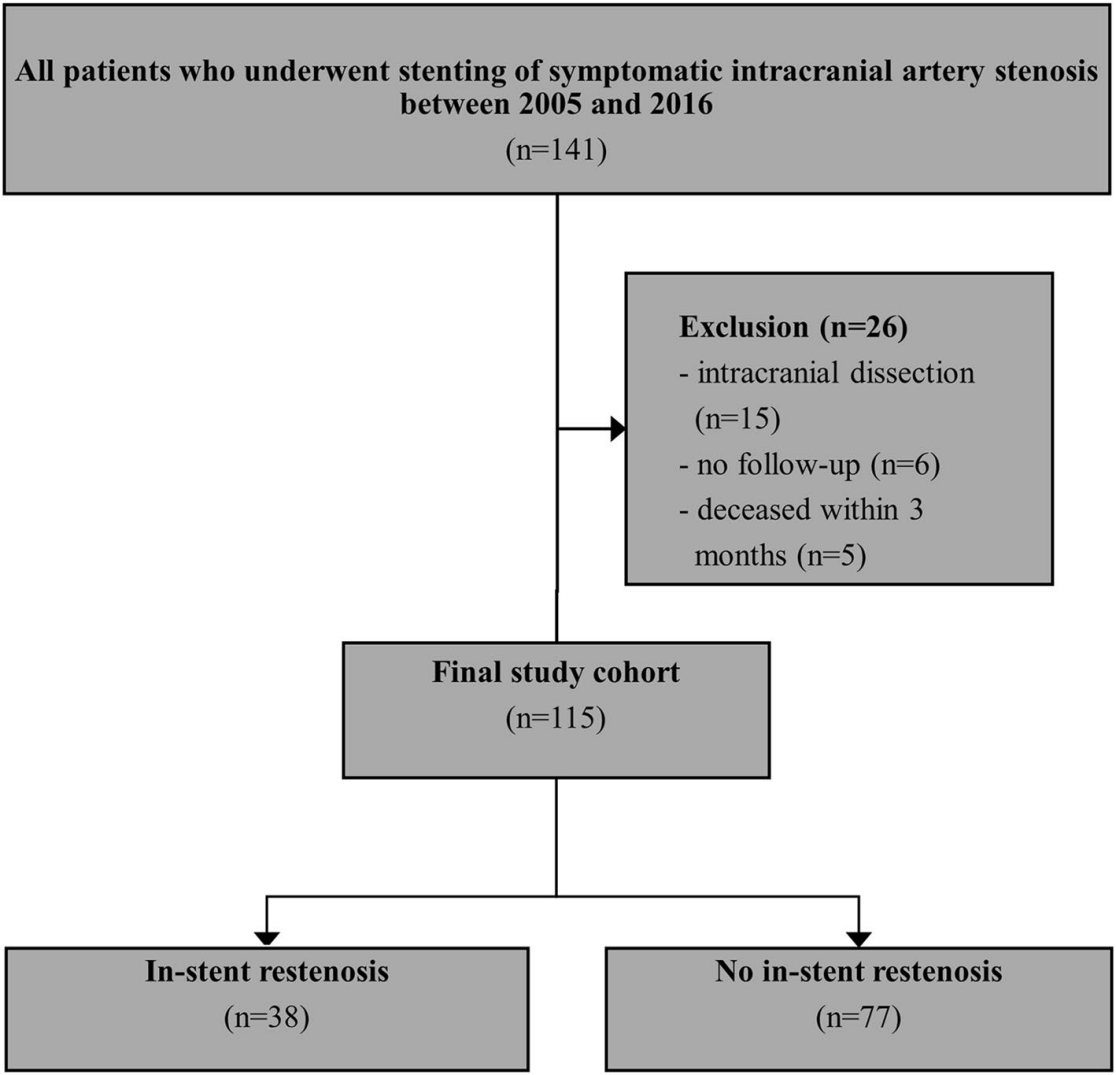
Flow diagram of patient selection.

This study was performed as a retrospective analysis, which included all patients who had undergone stenting of symptomatic high-grade ICAS at our primary and tertiary care university hospital between 2005 and 2016. Patients with ICAS related to a non-atherosclerotic etiology (e.g. intracranial dissection) were excluded (Fig. 1).

ICAS was diagnosed by transcranial Duplex sonography (TCD) and confirmed by cerebral magnetic resonance or computed tomography (CT) angiography. Stenting was performed in patients with symptomatic high-grade (≥ 70%) intracranial stenosis. A stenosis was deemed symptomatic, if the ischemic stroke corresponded to the territory of the affected artery. Stenting was not performed if the patient had severe concomitant disease such as malignant cancer, dementia, severe heart or lung disease or contraindications for postinterventional dual antiplatelet therapy. For these reasons, every case was individually discussed in an interdisciplinary board including experienced vascular neurologists and interventional neuroradiologists.

### Patient data assessment

Patient data at baseline including demographics, vascular risk profile as well as past medical history and comorbidities were obtained from our neurovascular stenting database and were completed using the electronic medical documentation system of our university hospital^17^. Vascular risk factors were defined according to recent guideline recommendations^18–22^ or if respective (e.g. antihypertensive) medication was already prescribed.

### Blood parameter assessment

Laboratory baseline examination was carried out 1 day before stenting and comprised a complete blood count analysis including MPV, lipid parameters, hemoglobin A1c, C-reactive protein, liver enzymes and renal function as well as coagulation parameters. Neutrophil to lymphocyte ratio (= quotient of absolute number of neutrophil granulocytes and lymphocytes) and platelet to lymphocyte ratio (= quotient of absolute number of thrombocytes and lymphocytes) were calculated retrospectively from originally obtained values. MPV was calculated by dividing the plateletcrit by the total number of platelets (reference range 7–13 fL).

### Stenting procedures

Stenting of high-grade ICAS was performed by neurointerventional specialists under local anesthesia via groin puncture. In most cases (97%) self-expanding Nitinol Wingspan stents (Boston Scientific, Natick, MA, USA) were used. Only three patients were treated with drug-eluting stents.

At the end of the intervention, control angiography was performed to detect a potential residual stenosis in the treated vessel. After the intervention, patients were monitored at the stroke unit or neuro-intensive care unit.

All patients received full-dose heparin at the day of the intervention and until 24 h post stenting according to our standard operating procedure. On the first postinterventional day cerebral magnetic resonance imaging (MRI) was performed in order to identify newly emerged cerebral infarcts. Dual antiplatelet therapy (DAPT, aspirin 100 mg and clopidogrel 75 mg daily) was started at least 3 days before stenting and continued for a minimum of 3 months after the intervention. Afterwards, DAPT was usually replaced by antiplatelet monotherapy (aspirin or clopidogrel). All patients received statin therapy and intense control of modifiable vascular risk factors. LDL target levels were set according to the most recent guideline recommendations within the study period (2005–2010: LDL < 100 mg/dL; since 2011: LDL < 70 mg/dL)^20,21^.

### Follow-up

All included patients underwent a standard follow-up regime comprising clinical examination and TCD at 1, 3 and 6 months, and annually after the stenting procedure. Six months after the intervention, digital subtraction angiography or cerebral CT angiography was routinely performed. In case of ISR detection on TCD during follow-up, additional CT angiography was performed for confirmation. Laboratory examinations were analysed when ISR was detected or at last clinical follow-up. ISR was defined as ≥ 50% restenosis in the affected vessel. Patients who did not participate in the follow-up examinations or had deceased within 3 months after the intervention were excluded from the study (n = 11, Fig. 1).

### Statistics

For statistical analysis, the IBM SPSS Statistics (Version 25) was used. Continuous variables are shown using mean and standard deviation (SD), nominal parameters are presented in absolute numbers and percentages. To compare nominal data, Pearson’s Chi-square test and Fisher’s exact test were used. For continuous variables, the Gaussian distribution was proven with the Kolmogorov–Smirnov test. In a further step, Student's *t* test (parametric data) or Mann–Whitney *U* test (non-parametric data) were used.

Blood biomarkers of interest (MPV, NLR, CRP, PLR) were additionally adjusted for age and sex in a binary multivariable logistic regression model for ISR as the target variable.

Statistical significance was defined as a probability value below 0.05.

### Ethics approval and consent to participate

The study was approved by the ethics committee of the Medical University of Graz (ethics committee number: 24-474 ex 11/12) and was carried out in accordance with the relevant guidelines and regulations. Informed consent was waived by the ethics committee because of the retrospective nature of the study.

## Results

Of 141 patients who had undergone stenting of symptomatic intracranial artery stenosis, 115 patients (mean age: 73 ± 13 years, 34% female) were included in the final study cohort (Fig. 1). Arterial hypertension (86%) and hyperlipidaemia (80%) were the most common vascular risk factors.

Digital subtraction angiography at the end of the intervention showed residual low-grade stenosis (< 50%) in seven individuals (6%). 10 patients (9%) had peri-interventional complications including arterial dissection (n = 3), subarachnoid hemorrhage (n = 4) and ischemic stroke (n = 3). All treated patients underwent repeated clinical and ultrasonographic follow-up investigations for a mean period of 68 months (SD: ± 43 months).

### In-stent restenosis

38 patients (33%) had ISR, which was detected in follow-up examinations after a mean period of 11 months (SD: ± 18 months). Of those, ISR was symptomatic in 7 patients (18%, all ischemic strokes). Common vascular risk factors did not differ between patients with versus without ISR (Table 1).

**Table 1:** Demographics, risk factors and laboratory parameters at baseline dichotomized according to the occurrence of in-stent restenosis after stenting of symptomatic intracranial artery stenosis.

	All patients (n = 115)	In-stent restenosis (n = 38)	No in-stent restenosis (n = 77)	P value
**Demographics**				
Women (%)	39 (33.9)	13 (34.2)	26 (33.8)	0.962
Age (years)	72.5 ± 13.1	74.5 ± 12.7	71.5 ± 13.2	0.258
**Risk factors (n, %)**				
Hypertension	99 (86.1)	35 (92.1)	64 (83.1)	0.190
Diabetes mellitus	47 (40.9)	13 (34.2)	34 (44.2)	0.308
Hyperlipidemia	92 (80.0)	33 (86.8)	59 (76.6)	0.198
Active smoking at baseline	28 (24.3)	9 (23.7)	19 (24.7)	0.907
Persistent smoking during follow up	12 (10.4)	4 (10.5)	8 (10.4)	0.982
Coronary artery disease	29 (25.2)	13 (34.2)	16 (20.8)	0.119
Peripheral artery disease	21 (18.3)	5 (13.2)	16 (20.8)	0.320
**Localization of intracranial stenosis (n, %)**				
Internal carotid artery	37 (32.2)	8 (21.1)	29 (37.7)	0.073
Middle cerebral artery	33 (28.7)	13 (34.2)	20 (26.0)	0.358
Vertebral artery	36 (31.3)	14 (36.8)	22 (28.6)	0.368
Basilar artery	30 (26.1)	10 (26.3)	20 (26.0)	0.969
Periinterventional complication (n, %)	10 (8.7)	3 (7.9)	7 (9.1)	0.830
**Hematological parameters at baseline (mean, SD)**				
Thrombocytes (× 10^9^/L)	224.2 ± 61.4	224.0 ± 52.7	224.3 ± 65.6	0.980
Leukocytes (× 10^9^/L)	7.0 ± 2.2	6.5 ± 1.5	7.3 ± 2.5	0.078
Neutrophils (× 10^9^/L)	4.7 ± 2.0	4.3 ± 1.3	4.9 ± 2.2	0.095
Lymphocytes (× 10^9^/L)	1.7 ± 0.6	1.6 ± 0.5	1.7 ± 0.6	0.658
Mean platelet volume (fL)	10.9 ± 1.0	10.8 ± 0.9	10.9 ± 1.1	0.729
Neutrophil to lymphocyte ratio (× 10^9^/L)	3.2 ± 2.0	3.0 ± 1.8	3.3 ± 2.1	0.439
Platelet to lymphocyte ratio (× 10^9^/L)	149.3 ± 69.7	154.2 ± 77.9	146.8 ± 65.6	0.595
**Biochemical parameters at baseline (mean, SD)**				
C-reactive Protein (mg/L)	11.5 ± 24.1	5.8 ± 7.6	14.4 ± 28.0	0.079
Creatinine (mg/dL)	1.0 ± 0.6	1.0 ± 0.3	1.1 ± 0.7	0.611
Hemoglobin A1c (mmol/mol)	47.6 ± 18.3	45.5 ± 18.6	48.7 ± 18.2	0.482
Low-density lipoprotein (mg/dL)	107.5 ± 43.4	106.7 ± 42.9	107.9 ± 44.0	0.899
Triglycerides (mg/dL)	168.4 ± 106.3	176.8 ± 91.1	163.9 ± 113.9	0.580
Recurrent ischemic event (n, %)	11 (9.6)	8 (21.1)	3 (3.9)	0.003

**Figure 2:**
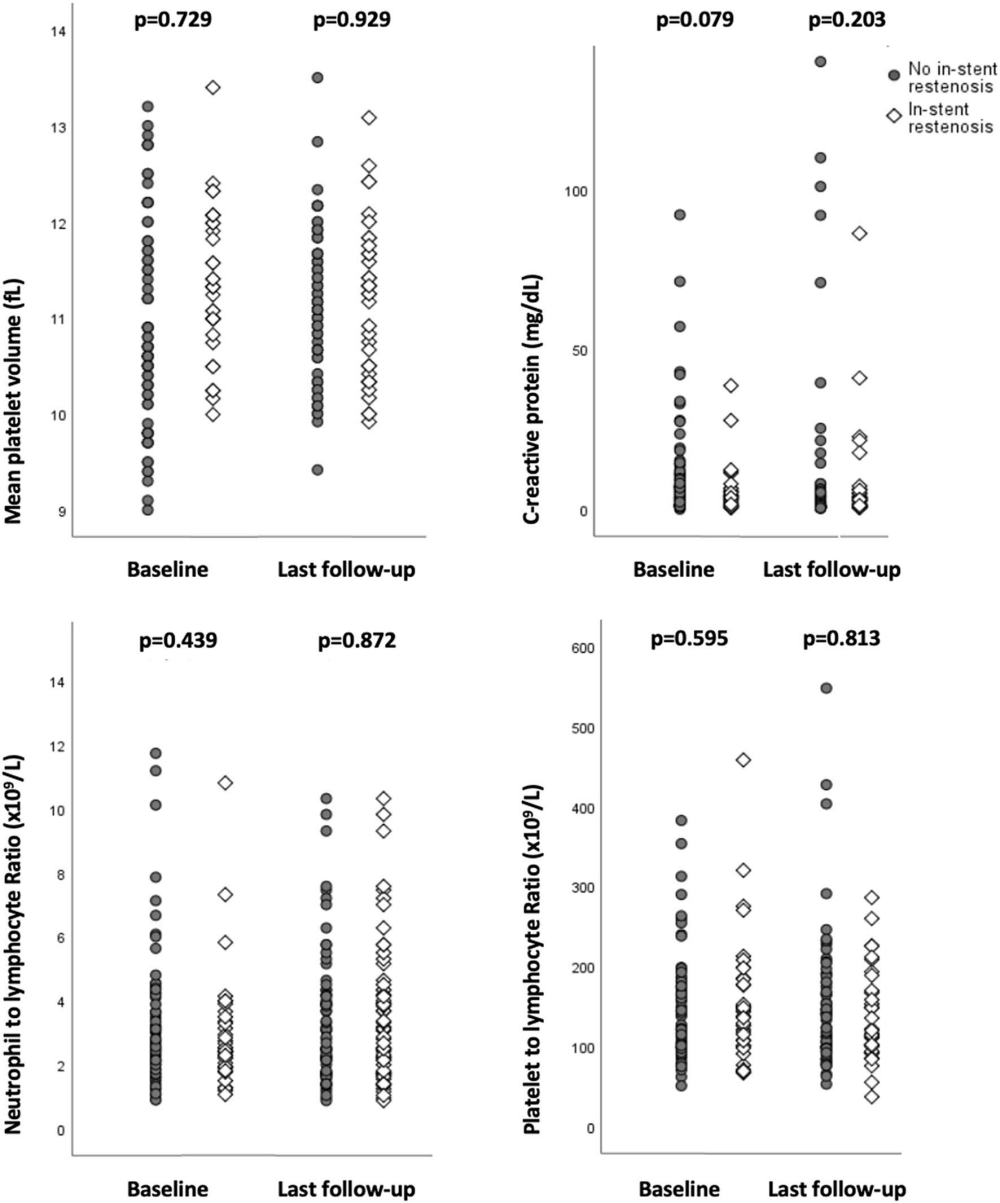
Blood biomarkers of platelet aggregation and inflammation in ISR and non-ISR patients after stenting of intracranial artery stenosis.

MPV values at baseline and at ISR-detection or last follow-up were comparable between ISR and non-ISR patients (p = 0.729 and p = 0.929, respectively) as were NLR (p = 0.439 and p = 0.872), PLR (p = 0.595 and p = 0.813) and CRP (p = 0.079 and p = 0.203) values (Fig. 2, Table 2). Moreover, all tested blood parameters did not change significantly from baseline to ISR-detection (p > 0.1). Multivariable regression analysis did also not identify significant predictors for ISR after stenting of ICAS (p > 0.1).

**Table 2:** Laboratory parameters at last follow-up dichotomized according to the occurrence of in-stent restenosis after stenting of symptomatic intracranial artery stenosis.

	All patients (n = 115)	In-stent restenosis (n = 38)	No in-stent restenosis (n = 77)	P value
**Hematological parameters at follow up (mean, SD)**				
Thrombocytes (× 10^9^/L)	234.0 ± 77.5	228.2 ± 66.1	236.8 ± 82.8	0.576
Leukocytes (× 10^9^/L)	8.0 ± 2.6	7.6 ± 2.6	8.1 ± 2.7	0.357
Neutrophils (× 10^9^/L)	5.3 ± 2.5	5.1 ± 2.4	5.4 ± 2.6	0.469
Lymphocytes (× 10^9^/L)	1.8 ± 0.7	1.7 ± 0.6	1.8 ± 0.7	0.435
Mean platelet volume (fL)	10.6 ± 0.9	10.6 ± 1.0	10.6 ± 0.9	0.929
Neutrophil/lymphocyte ratio	3.7 ± 3.2	3.6 ± 2.8	3.7 ± 3.4	0.872
Platelet/lymphocyte ratio	150.9 ± 90.4	153.7 ± 105.2	149.5 ± 82.5	0.813
**Biochemical parameters at follow up (mean, SD)**				
C-reactive protein (mg/L)	14.5 ± 36.1	8.1 ± 16.3	17.8 ± 42.7	0.203
Creatinine (mg/dL)	1.2 ± 0.7	1.1 ± 0.8	1.2 ± 0.7	0.628
Hemoglobin A1c (mmol/mol)	46.0 ± 12.9	45.5 ± 10.5	46.2 ± 14.0	0.838
Low-density lipoprotein (mg/dL)	88.6 ± 29.7	94.9 ± 33.1	85.4 ± 27.4	0.163
Low-density lipoprotein target level reached* (n, %)	28 (41.8)	20 (46.5)	8 (33.3)	0.294
Triglycerides (mg/dL)	149.7 ± 100.6	145.1 ± 60.1	152.4 ± 119.2	0.834

*Target levels were set according to recent guideline recommendations (2005–2010: LDL < 100 mg/dL; since 2011: LDL < 70 mg/dL).

### Recurrent ischemic cerebrovascular events

Of all included patients, 11 patients (10%) had a recurrent ischemic cerebrovascular event (stroke: n = 9, TIA: n = 2) after a mean follow-up period of 6 months (SD: ± 11 months).

Patients with ISR were more likely to suffer from stroke or TIA compared to the non-ISR group (11% versus 4%, p = 0.003). Stenting of middle cerebral artery stenosis (55% versus 26%, p = 0.046) was also significantly associated with recurrent cerebral ischemic events in univariable analyses. MPV, NLR, PLR and CRP at baseline and at last clinical follow-up were not predictive in this context (p > 0.1, Table 3).

**Table 3:** Demographics, vascular risk profile and laboratory parameters at baseline dichotomized according to the occurrence of recurrent ischemic cerebrovascular events after stenting of symptomatic intracranial artery stenosis during a 5-year follow-up.

	Recurrent ischemic stroke/TIA (n = 11)	No recurrent ischemic stroke/TIA (n = 104)	P value
**Demographics**			
Women (%)	5 (45.5)	34 (37.2)	0.395
Age (years)	71.9 ± 14.1	72.5 ± 13.0	0.880
**Risk factors (n, %)**			
Hypertension	10 (90.9)	89 (85.6)	0.627
Diabetes mellitus	5 (45.5)	42 (40.4)	0.745
Hyperlipidemia	10 (90.9)	82 (78.8)	0.342
Active smoking at baseline	2 (18.5)	26 (25.0)	0.616
Persistent smoking during follow up	0 (0.0)	12 (11.5)	0.234
Coronary artery disease	1 (9.1)	28 (26.9)	0.195
Peripheral artery disease	0 (0.0)	21 (20.1)	0.099
**Localization of intracranial stenosis (n, %)**			
Internal carotid artery	2 (18.2)	35 (33.7)	0.296
Middle cerebral artery	6 (54.5)	27 (26.0)	0.046
Vertebral artery	3 (27.3)	33 (31.7)	0.762
Basilar artery	3 (27.3)	27 (26.0)	0.925
Periinterventional complication (n, %)	0 (0.0)	10 (9.6)	0.282
**Hematological parameters at baseline (mean, SD)**			
Thrombocytes (× 10^9^/L)	208.7 ± 44.3	225.8 ± 62.9	0.382
Leukocytes (× 10^9^/L)	6.8 ± 1.9	7.1 ± 2.6	0.699
Neutrophils (× 10^9^/L)	4.4 ± 1.8	4.7 ± 2.0	0.568
Lymphocytes (× 10^9^/L)	1.7 ± 0.7	17. ± 0.6	0.869
Mean platelet volume (fL)	10.7 ± 0.9	10.9 ± 1.1	0.471
Neutrophil to lymphocyte ratio	3.0 ± 2.0	3.2 ± 2.1	0.806
Platelet to lymphocyte ratio	148.7 ± 79.1	149.3 ± 69.1	0.978
**Biochemical parameters at baseline (mean, SD)**			
C-reactive Protein (mg/L)	3.9 ± 2.1	12.4 ± 25.3	0.269
Creatinine (mg/dL)	0.9 ± 0.2	1.1 ± 0.6	0.403
Hemoglobin A1c (mmol/mol)	48.9 ± 23.4	47.5 ± 17.8	0.841
Low-density lipoprotein (mg/dL)	117.8 ± 69.7	106.3 ± 39.6	0.431
Triglycerides (mg/dL)	195.6 ± 102.1	165.1 ± 106.9	0.394
In-stent restenosis (n, %)	8 (72.7)	30 (28.9)	0.003

## Discussion

In this retrospective analysis, routine blood biomarkers that have been associated with progressive atherosclerosis in different vascular beds in other studies were not predictive for ISR or recurrent ischemic cerebrovascular events after stenting of symptomatic high-grade intracranial artery stenosis. In line with earlier studies^2,9,10^, ISR was a significant risk factor for the occurrence of recurrent stroke or TIA during a long-term follow-up period of up to 5 years, while tested blood biomarkers were not contributory.

Recently, routine blood biomarkers of inflammation and platelet aggregation were investigated regarding their predictive value for progressive arterial disease. MPV is considered a marker for platelet activity as larger platelets express denser granules, leading to a higher potential of platelet aggregation, neointimal hyperplasia and thrombo-embolism^23,24^. In this context, MPV has been associated with progressive atherosclerosis of coronary and peripheral arteries^12,24,25^. After carotid artery stenting, MPV has been identified as predictor for ISR in an Asian study population, but this could not be confirmed in a recent investigation in a larger European patient cohort^12,17^.

Inflammatory processes play a central role in all stages of atherosclerosis. CRP and NLR are indicators of increased local and systemic inflammation and have been related to more instable atherosclerotic plaques and progressive stenosis of intracranial arteries^13,26,27^. As reactive thrombocythemia is a well-known response on acute-phase reactions, it was not surprising that PLR has also been identified as independent predictor of progressive (coronary) artery disease^12,13^. All those blood biomarkers are easily available in clinical practice and could thus be valuable parameters to identify patients at risk for ISR or recurrent stroke after stenting of ICAS.

To the best of our knowledge, we here present the first study that investigated the predictive value of routine blood biomarkers for ISR after intracranial artery stenting over a long-term follow-up period of up to 5 years.

During the follow-up period, one third of all patients was diagnosed with ISR, which is comparable to recently published data (22–46%)^2,8,9^. However, we were not able to detect an association between tested blood biomarkers and ISR after stenting of ICAS neither at baseline nor at follow-up or ISR detection. Moreover, biomarker levels did not change from baseline to ISR-detection.

This contrasts with earlier studies that found an association between increased CRP and the occurrence of ISR after stenting of extra-/intracranial arteries and progressive intracranial large artery disease^27–30^. However, those studies were limited by a very small number of ICAS patients (n = 16)^28^ and did not differentiate between intra- and extracranial artery stenosis or excluded patients after intracranial artery stenting^29,30^. Moreover, these studies investigated relatively young study populations with a moderate number of classical vascular risk factors^29,30^. This might also explain the higher CRP levels detected in our study, as CRP is a non-specific marker of inflammation and acute-phase reaction, which increases with age and comorbidities^31^, and therefore might limit the value of CRP as a predictive marker for ISR after stenting.

Previous studies suggested an association between MPV and ISR after carotid and coronary artery stenting^14,15^. However, in those studies, patients who developed ISR had a high number of concomitant vascular risk factors (e.g. diabetes mellitus). It is known that arterial hypertension, diabetes mellitus and hyperlipidemia independently increase MPV levels^14^. This might explain the missing link between MPV and ISR after ICAS in this study, as vascular risk factors were comparable between ISR and non-ISR patients, which is further supported by two recent negative studies on the value of MPV on progressive carotid artery disease^17,32^.

Although our study is the largest investigation on ISR after stenting of ICAS to date, the number of included patients is considerably lower than those in recent studies that have investigated the value of NLR and PLR on progressive atherosclerotic disease (n > 1000) in other blood vessels^12,15^. Therefore, a weak correlation between NLR/PLR levels and ISR after stenting of ICAS could have been missed in our study. Nevertheless, we were able to exclude a major contribution of such markers to progressive intracranial artery disease, as we had laboratory studies at baseline and follow-up available.

Despite a long-term follow-up period of up to 5 years, which is notably longer compared to earlier studies focusing on ISR development after ICAS stenting^28,33^, we observed a low number of recurrent cerebrovascular events. The missing link between the tested blood biomarkers and recurrent stroke/TIA must therefore be interpreted cautiously. Another limitation of this study is its retrospective design as other possibly valuable biomarkers, that have been associated with progressive atherosclerosis in recent studies (e.g. lipoprotein a or lipoprotein associated phospholipase A2)^34–36^, were not available because they were not part of our routine laboratory examination. Nevertheless, the findings of this study render a major contribution of routine blood biomarkers of progressive atherosclerosis to the prediction of ISR after stenting of intracranial artery stenosis over a long-term 5-year follow-up period unlikely.

## Data Availability

Data are available from the corresponding author upon reasonable request.
